# Comparison of linear frequency and amplitude modulation for intraneural sensory feedback in bidirectional hand prostheses

**DOI:** 10.1038/s41598-018-34910-w

**Published:** 2018-11-12

**Authors:** G. Valle, F. M. Petrini, I. Strauss, F. Iberite, E. D’Anna, G. Granata, M. Controzzi, C. Cipriani, T. Stieglitz, P. M. Rossini, A. Mazzoni, S. Raspopovic, S. Micera

**Affiliations:** 1 Bertarelli Foundation Chair in Translational Neuroengineering, Centre for Neuroprosthetics and Institute of Bioengineering, School of Engineering, École Polytechnique Fédérale de Lausanne (EPFL), Lausanne, Switzerland; 2 Center for Neuroscience, Neurotechnology, and Bioelectronic Medicine and BioRobotics Institute, Scuola Superiore Sant’Anna, Pisa, Italy; 3 Institute of Neurology, Catholic University of The Sacred Heart, Policlinic A. Gemelli Foundation, Roma, Italy; 4 Laboratory for Biomedical Microtechnology, Department of Microsystems Engineering–IMTEK, Bernstein Center, BrainLinks-BrainTools Cluster of Excellence, University of Freiburg, Freiburg, D-79110 Germany; 5 Department of Health Sciences and Technology, Institute for Robotics and Intelligent Systems, ETH Zürich, 8092 Zürich Switzerland

## Abstract

Recent studies have shown that direct nerve stimulation can be used to provide sensory feedback to hand amputees. The intensity of the elicited sensations can be modulated using the amplitude or frequency of the injected stimuli. However, a comprehensive comparison of the effects of these two encoding strategies on the amputees’ ability to control a prosthesis has not been performed. In this paper, we assessed the performance of two trans-radial amputees controlling a myoelectric hand prosthesis while receiving grip force sensory feedback encoded using either linear modulation of amplitude (LAM) or linear modulation of frequency (LFM) of direct nerve stimulation (namely, bidirectional prostheses). Both subjects achieved similar and significantly above-chance performance when they were asked to exploit LAM or LFM in different tasks. The feedbacks allowed them to discriminate, during manipulation through the robotic hand, objects of different compliances and shapes or different placements on the prosthesis. Similar high performances were obtained when they were asked to apply different levels of force in a random order on a dynamometer using LAM or LFM. In contrast, only the LAM strategy allowed the subjects to continuously modulate the grip pressure on the dynamometer. Furthermore, when long-lasting trains of stimulation were delivered, LFM strategy generated a very fast adaptation phenomenon in the subjects, which caused them to stop perceiving the restored sensations. Both encoding approaches were perceived as very different from the touch feelings of the healthy limb (natural). These results suggest that the choice of specific sensory feedback encodings can have an effect on user performance while grasping. In addition, our results invite the development of new approaches to provide more natural sensory feelings to the users, which could be addressed by a more biomimetic strategy in the future.

## Introduction

In the recent past, a major effort has been dedicated to the use of implantable peripheral interfaces to stimulate the residual nerves of upper limb amputees in order to restore sensations in their phantom hand^1–12^. Different encoding strategies have been used to translate the readout of sensors embedded or added into the prosthesis into stimulation parameters (namely, the amplitude, the pulse width, the repetition frequency and the duration of biphasic pulse trains).

Direct neural stimulation has restored sensory feedback and enabled the users to improve prosthesis control^4,7,8^, reduced phantom limb pain^9^, and increased the perception of the device as part of the body (embodiment)^4,13^. In these cases, sensory feedback has mainly relied on two encoding strategies for sensory feedback, which are the linear modulation of either the charge^3,5,7,9^ or the frequency^1,3,6,8,10^ of the stimulus pulses.

A recent study, performed on two trans-radial amputees, showed that both the direct neural modulation of charge (i.e., the amplitude or the pulse duration) and of frequency similarly controlled the intensity of the evoked sensations reported by the subjects^3^. This result is in accordance with the physiology of afferent fibres (according to the population model^14,15^), which deliver information about the intensity of a sensation to the brain through population recruitment (i.e., more spiking fibres) or changes in firing activity^14,16,17^. Recruitment and firing activity are controlled by the modulation of the amplitude and frequency of stimulation, respectively.

**Figure 1:**
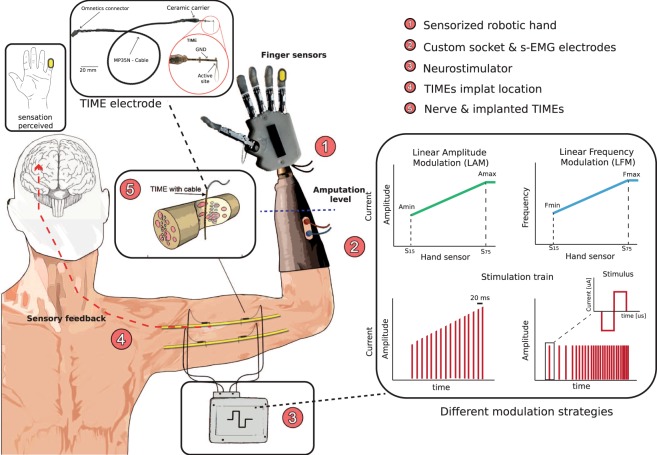
Bidirectional hand prosthesis. The upper limb amputees wore a robotic prosthesis equipped with a system for restoring sensory feedback through intraneural stimulation conveyed by TIME electrodes. The prosthesis was equipped with a custom socket and 2 force sensors. The readout from these sensors was transmitted to an external controller, which transduced it in stimulation parameters, modulating linearly the amplitude (LAM, average amplitude 150 ± 57.7 μA for Subject 1 and 250 ± 57.7 μA for Subject 2) or the frequency (LFM, average frequency 30 ± 23.1 Hz for Subject 1 and Subject 2) of the stimulation trains being injected. These instructions drove the activity of an external stimulator, which was connected to four TIME electrodes, previously implanted in the median and ulnar nerves. The neural interfaces were inserted transversally in the nerve, penetrating the fascicles.

Despite these achievements, the effect of the choice of encoding strategies on the performance of the amputee when controlling a prosthesis has not been investigated. To investigate this matter, we implanted two upper limb amputees with four TIME electrodes^18^, two in the median and two in the ulnar nerve, and used sensory feedback stimulation while linearly modulating either its frequency or amplitude (Fig. 1). We compared the effects of these two encoding strategies on the location, extent, and intensity of perceived sensations. We also measured one aspect of the adaptation to direct nerve electrical stimulation, i.e., the loss of perceived sensation after sustained stimulation, associated with the two strategies^19,20^. Then, we compared the ability of the subjects to control the bidirectional hand prosthesis during the execution of functional tasks.

## Results

### Sensation characterization

We first characterized the subjects’ rating of the stimulation delivered through TIMEs. We injected biphasic trains of current pulses lasting 2 s, using linear amplitude modulation (LAM) or linear frequency modulation (LFM), through each of the 14 active sites of the electrodes. For the amplitude modulation, the pulse amplitude varied between 10 µA and 980 µA, and the pulse frequency was fixed at 50 Hz^15^. For the frequency modulation, the pulse amplitude was fixed at the perceptual threshold value, and the pulse frequency was modulated between 1 to 1000 Hz. Interestingly, we discovered that selecting the minimum amplitude eliciting a perception threshold at 50 Hz and then diminishing the frequency down to zero caused a loss of sensation.

**Figure 2:**
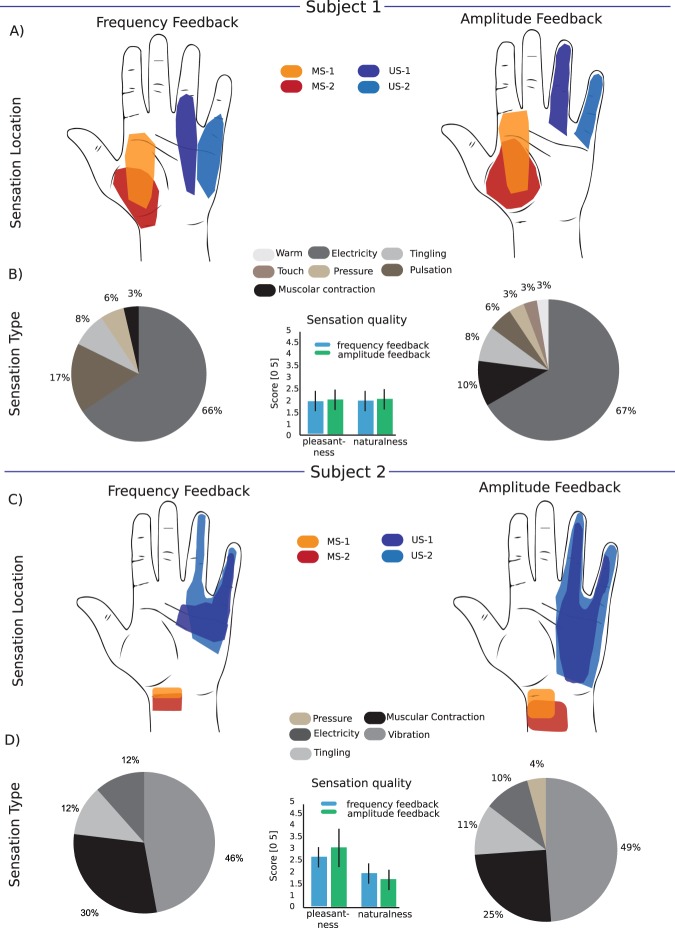
Location and quality of evoked sensations. (**A,C**) Maps of hand sensations with the four active sites (AS) tested for each subject for amplitude (right) and frequency (left) encoding. Each coloured area indicates the 75^th^ percentile of all the phantom sensations evoked for an AS during the all trial. The maps are related to each implant and are generated by the mapping procedure. For Subject 1, MS-1 (orange area) and MS-2 (red area) were the electrodes #1 AS1 and #2 AS4 implanted, respectively, in the proximal and distal parts of the median nerve; US-1 (blue area) and US-2 (light blue area) were the electrodes #3 AS 6 and #4 AS8 implanted, respectively, in the proximal and distal parts of the ulnar nerve. For Subject 2, MS-1 (orange area) and MS-2 (red area) were the electrodes #1 AS 7 and #2 AS 14 implanted, respectively, in the proximal and distal parts of the median nerve; US-1 (blue area) and US-2 (light blue area) were the electrodes #3 AS 12 and #4 AS 13 implanted, respectively, in the proximal and distal parts of the ulnar nerve. (**B,D**) Distribution of the sensation type for each subject according to all the phantom sensations evoked by all the AS using both encoding strategies. The bar plots show the qualities of the sensation evoked on a scale from 0 to 5 reported by the subjects during each repetition of the mapping procedure using LAM or LFM in terms of naturalness and pleasantness for the four AS (N = 10 repetitions x 4 AS = 40 for Subject 1 and N = 10 repetitions x 4 AS = 40 for Subject 2). The two-tailed Kruskal-Wallis test did not show a significant difference in the sensation quality between amplitude and frequency modulation in either subject (p > 0.05).

The subjects were asked to report the location, extent, type and quality and the strength of the sensation (rated from 0 to 10). About quality, we asked the subjects to rate naturalness (how much the sensation resembles those perceived with the intact limb) and pleasantness, on a scale from (fully unnatural) 0 to 5 (fully natural). Sensation qualities were asked at the perceptual threshold. Two active sites on the electrodes implanted in the median nerve and two active sites on the electrodes implanted on the ulnar nerve (Fig. 2A,C) were selected to restore sensory feedback in the closed loop of the bidirectional prosthesis. Here, we report the results from the characterization of their stimulation. The whole results of the sensation characterization on 56 AS of both subjects is reported in Petrini *et al*.^21^.

#### Location, type, and quality of the evoked sensation

For Subject 1, the stimulation sites in the median nerve elicited sensations in overlapping areas of the thenar eminence when the frequency or the amplitude modulation were used (Fig. 2A). The stimulation sites in the ulnar nerve elicited sensations in areas close to the basis of the ring finger and little finger with LFM and on those two fingers with LAM. The kinds of sensation elicited by LAM and LFM were very similar, with a predominance of *electricity* (Fig. 2B). Sensation qualities did not significantly differ across the two modalities: pleasantness was rated 2 ± 0.5 with LFM and 2.1 ± 0.7 with LAM (p = 0.597 and d = 0.16), and naturalness 2 ± 0.3 and 2.2 ± 0.3 (p = 0.446 and d = 0.67), respectively (Fig. 2B, inset).

For Subject 2, the active sites in the median and ulnar nerves elicited sensations close to the wrist and to the ring and little fingers, respectively (Fig. 2C). The kinds of sensation elicited by LAM and LFM were similar for Subject 2, albeit with a predominance of *vibration* (Fig. 2D). Pleasantness and naturalness were not significantly different: pleasantness was 2.6 ± 0.3 with LFM and 3 ± 1.1 with LAM (p = 0.331 and d = 0.50), and naturalness 1.9 ± 0.4 and 1.6 ± 0.4 for LFM and LAM (p = 0.063 and d = 0.75), respectively (Fig. 2D, inset).

#### Extent and strength

**Figure 3:**
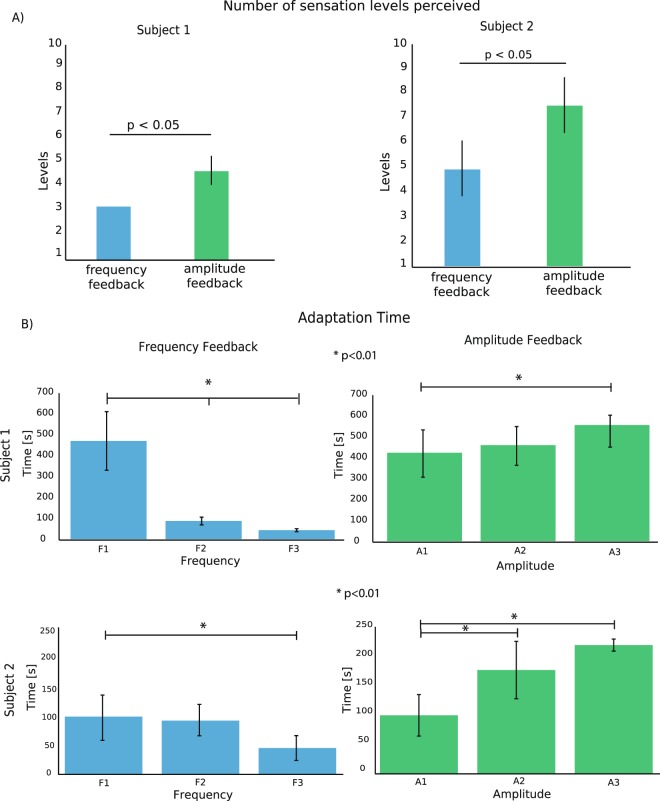
Perceived sensation intensities and adaptation. (**A**) The bar plots represent the number of significantly different pressure levels perceived by the subjects for all AS (related to N = 5 repetitions x 4 AS = 20 for each stimulation condition). Two-tailed Kruskal-Wallis test was performed (p < 0.05). (**B**) The adaptation time is shown at different frequencies and amplitudes of the stimulation trains tested with both subjects. A1, A2, and A3 or F1, F2, and F3 are the amplitudes or frequencies, respectively, at which the subjects reported perceptual threshold (A1, F1), medium intensity (A2, F2) and high intensity (A3, F3) of the sensation perceived during the mapping procedure. F1 = 10–50 Hz, F2 = 200–350 Hz, F3 = 700–800 Hz, A1 = 100–200 µA, A2 = 250–350 µA, A3 = 400–500 µA, depending on the AS, for Subject 1; F1 = 10–50 Hz, F2 = 150–250 Hz, F3 = 400–500 Hz, A1 = 200–300 µA, A2 = 350–450 µA, A3 = 500–600 µA, depending on the AS, for Subject 2. The data in the figure are represented as the mean ± std. No error bar on LFM for subject 1 in panel A is shown, since in all the repetitions the number of different sensation levels were always 3. *Indicates p < 0.01. The two-tailed Kruskal-Wallis test with Tukey-Kramer correction for multiple groups of data was performed. N = 3 frequencies or amplitudes x 20 repetitions = 60 for both subjects for each condition (LAM and LFM).

As expected, the linear increase in amplitude and frequency stimulation led to an increase in the intensity of the perceived sensation (Fig. 3A, Figure S1 and Figure S7). The number of significantly differently perceived levels of intensity was for both subjects higher with LAM than with LFM (4.75 ± 0.5 vs 3 ± 0.0 for all four electrodes for Subject 1; 6.25 ± 1 vs 3.75 ± 1 for Subject 2; Kruskal-Wallis test, p < 0.05 for both subjects, Fig. 3A). In both subjects, we observed a small enlargement of the area of the evoked sensation during LAM (Figure S2), as seen by Tan *et al*., 2014^9^. As expected^14^, the area increase was not observed when subjects were stimulated using LFM.

#### Adaptation

To evaluate the time at which the evoked sensation ceased to be perceived by the subject, we designed the following protocol. A train of pulses having constant amplitude, duration and frequency was delivered. The subjects were thus asked to report the exact moments at which the sensation decreased and then disappeared. Three different frequencies and amplitudes were tested, which elicited minimum-, medium-, and maximum-intensity sensations.

When we tested LFM, we found that adaptation time was prolonged only when low frequency pulses were delivered (Fig. 3B): adaptation time for Subject 1 ranged from 460 ± 101 s for low frequencies to 40 ± 12 s for high frequencies, a 91% reduction (Kruskal-Wallis, p < 0.01). For Subject 2, it ranged from 100 ± 48 s for low frequencies to 47 ± 14 s for high frequencies, a 53% reduction (Kruskal-Wallis, p < 0.01).

LAM displayed a slower adaptation than LFM. In particular, the adaptation time increased with the stimulation amplitude and therefore with the perceived intensity. For Subject 1, there was a significant (Kruskal-Wallis, p < 0.01) increase of +32% in adaptation time from 400 ± 90 s for the minimal intensity to 530 ± 30 s for the strongest intensity. Similarly, for Subject 2, adaptation ranged from 98 ± 42 s to 200 ± 20 s, a 104% increase (Kruskal-Wallis, p < 0.01).

Over the whole range of intensities and frequencies, in Subject 1 LAM produced an average adaptation time of 475 s versus 198 s during LFM, while Subject 2 exhibited an average adaptation time of 160 s and 87 s for LAM and LFM, respectively.

Moreover, while the dynamics across subjects was only qualitatively similar (the adaptation time constants were different from patient to patient, as in Graczyk *et al*.^20^), the adaptation times associated with different sites for the same subject were extremely similar (Figure S9).

### Functional tasks performance

**Figure 4:**
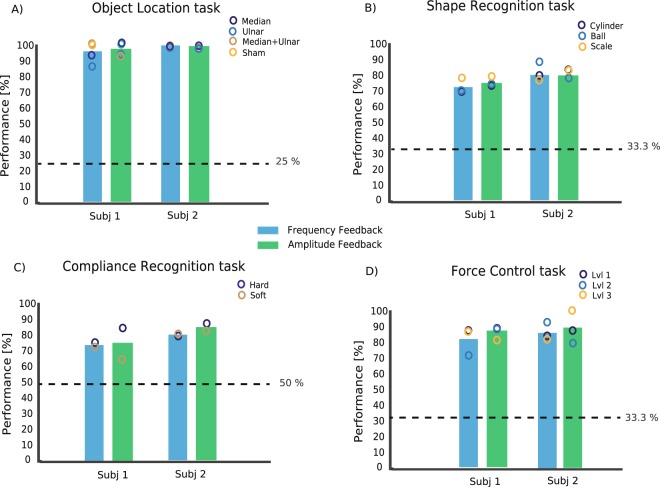
Performance in functional tasks. (**A**) Performance in the Object Location Task, (**B**) Shape Recognition Task, (**C**) Compliance Recognition Task and (**D**) Force Control Task for all the subjects in amplitude and frequency modulation modalities. The circles indicate the partial performance for each condition (e.g., for object recognition, the performances in recognizing the location on the median area, the ulnar area, or both). The dashed lines show the chance levels. In every functional task, N = 90 repetitions were performed for each subject in each condition. Fisher’s exact test was used to test the difference between amplitude and frequency modulation performance. No significant difference in the performance was found (p > 0.05).

Once the characteristics associated with the sensation elicited by each stimulation site were determined, we tested the effects of the two encoding strategies on the performance of five functional tasks: Object Location, Compliance and Shape Recognition, Force Control and Staircase Force Control. In the *Object Location Task* (OLT) the subjects were asked to grasp an object with the robotic hand and to recognize three positions in which a plastic cylinder was placed (ulnar, median zone or both). They identified the stimulation site (including sham) almost perfectly (Fig. 4A) with both encoding strategies, with a uniform performance for the four options. Subject 1’s performance was 95.5% for LFM and 97% for LAM (Fisher’s exact test p > 0.05) while Subject 2 performed 100% with both strategies.

In the *Shape Recognition Task* (SRT), they had to distinguish three object shapes (ball, cylinder and trapeze). The encoding strategy had no significant effect on the performance (Fisher’s exact test, p > 0.05): 73.3% and 76.7% with LFM and LAM, respectively for Subject 1, and both 83.3% for Subject 2 (Fig. 4B).

In the *Compliance Recognition Task* (CRT), the subjects had to distinguish two different object compliances (soft, hard). The two encoding strategies performed similarly in this test (Fisher’s exact test, p > 0.05): 75% using LFM and 73% using LAM for Subject 1, 80% for LFM and 85.5% for LAM for Subject 2 (Fig. 4C).

In the *Force Control Task* (FCT), the subjects were asked to apply three levels of grasping force (low, medium, and high) on a dynamometer, relying only on the intraneural stimulation. In particular, the subjects had to exert a single grasp at the required level of force, maintain it for approximately 2 seconds, and then release the grip. The subjects were able to consistently modulate the grip force at the three different levels (Fig. 4D). The performance with LFM and LAM was not significantly different (Fisher’s exact test, p > 0.05): 82.3% and 87% respectively for Subject 1, and 87% and 88.3% for Subject 2 (Fig. 4D).

**Figure 5:**
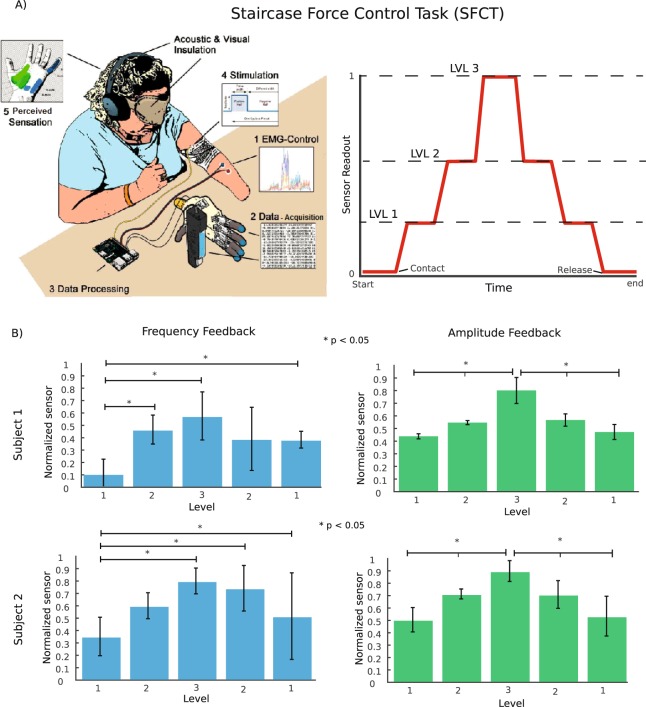
Staircase force control task. (**A**) Schematic representation of the staircase force control task. The right panel displays the normalized sensor outputs required by the subject. The subjects were asked to perform three force levels in the rising phase and two in the releasing phase. (**B**) Statistical analysis and reproducibility of the force output for the robotic hand finger. The Kruskal-Wallis test with the Tukey-Kramer post hoc test for multi-group comparison was performed over all the single-step force trials (N = 5 levels x 10 repetitions x 2 conditions for Subject 1 and N = 5 levels x 10 repetitions x 2 conditions for Subject 2). The test shows three significantly different levels achieved in terms of the maximum force per level reached using amplitude. The data in the figure are represented as the means ± std. *Indicates p < 0.05.

In the *Staircase Force Control Task* (SFCT), the participants were asked to gradually increase the applied force in three levels and then gradually return to the baseline. The subjects were asked to maintain each level of pressure until they felt confident with the exerted level of force. A schematic representation of the task is shown in Fig. 5A. The two subjects performed the task similarly. The staircase task with LFM failed (Fig. 5B, left column): the force associated with level 2 was never significantly different from the force associated with level 3 (p > 0.1 for both subjects and both rising and releasing phases, Kruskal-Wallis test with Tukey-Kramer post hoc correction for multiple comparisons). Both subjects instead managed to perfectly accomplish the task when presented with LAM (Fig. 5B, right column). Each level was significantly different from the previous and the next one (p < 0.01) and not significantly different between the rising and releasing phases (p > 0.1).

Confusion matrices for all functional tasks, with single subjects, are shown in Figure S3–S4. It is worth noting that OLT, SRT, CRT and FCT results were stable over time, as the performance of the two subjects in all four functional tests repeated one month after the first session showed no significant difference (Fisher’s exact test, p > 0.05) (Figure S5).

## Discussion

Two complementary encoding strategies, based on the linear modulation of amplitude or frequency according to the signals recorded from artificial sensors, were implemented using intraneural electrodes and tested during several functional tasks. We found that in many respects, the two encoding strategies performed in a similar way, but the amplitude modulation was able to convey more sensitive and reliable tactile feedback overall.

### Similarities between LAM and LFM

The two encoding strategies elicited very similar sensations in both subjects (Fig. 2B,D), as these are probably determined by the location of the stimulation site. The quality of the sensations was also the same (Fig. 2B,D insets). In particular, for both strategies, both naturalness and pleasantness were very far from a satisfying level, suggesting that different strategies must be used to achieve a naturalistic and more pleasant sensation.

The two encoding strategies allowed the two subjects to achieve very similar performance during all the functional tests (Fig. 4). In particular, they performed in a very similar way in locating the stimulation, in recognizing the shape of three objects of similar size, in discriminating hard and soft compliance and in controlling three different levels of force. In all these tasks, the subjects were asked to discriminate items, relying on sensations elicited by short trains of stimulation. It is safe to conclude that for tasks involving short-lasting stimulations, the two encoding strategies transmit the same amount of useful information to the subjects.

### Differences between linear encoding of amplitude and frequency

The first difference we spotted between the two strategies was the extent of the elicited sensation: as already reported in^3,9^, LAM was associated with a modulation of the percept area, while LFM was not (Figure S2). This might be because amplitude modulation is correlated to the number of recruited fibres, while frequency modulation is not^9,15,22–24^. Functional consequences of this difference are complex and hard to evaluate. This shows that in single contact stimulations through LAM, the sensation intensity and sensation area are not independent. This represents a constraint towards the implementation of completely naturally perceived restored sensory feedback, since the intensity and area of the sensation are independent in the intact hand: in normal conditions we are able to perceive an intense and very localized pressure as well as a gentle pressure over the whole palm, while using LAM feedback an intense pressure is always associated with pressure being perceived over a larger area. At the same time, the simultaneous increases in the sensation area and perceived intensity make strong pressures easier to detect using LAM feedback.

The second difference was that in both subjects the number of perceived levels was higher with LAM than with LFM (Fig. 3A). This allows for the encoding of a broader set of sensory intensities associated with corresponding levels of applied pressure^7,25^, leading to a higher information capacity for LAM than for LFM. Our results are in line with those found previously by using extraneural electrodes (FINE)^20^ and transcutaneous electrical nerve stimulation (TENS)^26^. In both of those studies, indeed, although the nerve interfaces were different, charge modulation allowed to the subjects to have a better stimulus discriminability than when frequency modulation was adopted. Here, this was shown by the different number of perceived levels between the two modulation strategies.

Furthermore, we observed that the actual difference between LAM and LFM in the range of elicited intensity levels, exploitable in real-life situations, might be even broader due to adaptation^27,28^: prolonged stimulations with varying frequencies are not possible, since adaptation causes the sensation to fade in less than a minute, while all amplitude intensities elicited a response that lasted more than a minute and half. We envisage two possible reasons why the subjects were able to complete the SFCT only with amplitude encoding. The first is the differences in adaptation time. Indeed, the time to finish this task exceeded one minute (Fig. 5 and Supplementary Table 1). The second one is that too few discriminable intensity levels were provided with LFM to enable the subjects to successfully accomplish the task. However, we can rule out the second hypothesis because, as shown in Fig. 4 and Figure S10, LFM allowed them to discriminate the three levels of force in FCT and in SFCT when presented in ascending order. Therefore, we might hypothesize that adaptation is the reason the two encoding strategies gave different results in the Staircase Test.

A recent study showed that the nerve sensibility decreases for constant (e.g., fixed frequency and charge) direct nerve stimulation^20^, but the sensation does not disappear. They considered, though, a lower frequency range (<200 Hz) and investigated shorter intervals (<3 minutes). The failure of the transmission of high-frequency stimulations is unlikely to be caused by failure in nerve conduction, since nerve block normally occurs at frequencies higher than 2 kHz^29,30^. Mechanoreceptors cannot be the cells in which sensory adaptation takes place in this case, since the neural stimulation bypasses the mechanoreceptors^20^. We suggest that synaptic mechanisms or an increase in spike generation threshold, which is commonly facilitated by an increase in the stimulation frequency, could be the reasons^19,31–34^. This phenomenon is likely due to supra-peripheral factors (i.e., spinal cord synapses or medulla).

Regarding the results achieved with LAM, we hypothesize that since the recruitment of neurons increases because of the higher stimulation amplitudes^15,22,23^, more synaptic failures have to occur for the elicited sensations to disappear.

### Advantages of the amplitude modulation approach in a bidirectional prosthesis

We found that the intensity of the sensations was more stable with LAM than with LFM, i.e., the adaptation time is longer over the whole range of intensities available. This suggests that in real-life situations involving a sustained feedback evolving over time, LAM might be preferable to LFM. Indeed, we found that the subjects managed to accomplish the staircase force control task only by means of amplitude modulation (Fig. 5). With LFM it was difficult to detect slow variations in the feedback (no subject discriminated between levels 2 and 3), and the sensation was history dependent: equivalent levels in the rising and releasing phases were felt as different due to the different sequence of states preceding them. With LAM, every level of feedback was clearly perceived as different from the others and perceived as identical between the rising and releasing phases. It is possible that the higher capability achieved through LAM to continuously modulate different force levels over time is connected to the increased sensitivity described in Figs 3A and S6.

Overall, our results suggest that linear amplitude modulation might be preferable to linear frequency modulation for controlling hand prostheses. Note that this conclusion does not rule out the possibility that more complex feedback-encoding strategies exploiting frequency modulation, such as the neuromorphic strategy used in^11^ or the Touch-Stim model in^35,36^, might be able to perform even better than linear amplitude encoding.

### Limitations and perspectives

The main limitation of the present work is that we restrained our investigation to linear modulation of amplitude and frequency as a function of the stimulus intensity. As briefly mentioned above, more complex feedback strategies could be envisaged, such as neuromorphic stimulations^11,35,37,38^ mimicking more closely the physiological behaviour of natural sensors in the skin. Following this approach, the frequency of the stimulation is modulated according to the firing rate of spike trains produced by models injected with an input current proportional to the pressure^11^ and its derivatives^36^, mimicking the behaviour of natural mechanoreceptors^39^. In the future, it would be intriguing to extend the comparative approach used in the present work to include these approaches.

The very first studies on upper limb prosthesis control^40^ identified naturalness as a desired feature^41^. In this respect, LAM and LFM were equivalent in our tests (see Fig. 2). In future works, we will investigate how to evoke close-to-natural sensations while preserving or increasing the efficacy of the encoding^36–38^.

Moreover, the purpose of this study was to compare the feedback from subjects according to the specific encoding algorithms for bidirectional prostheses. The adaptation experiment was only conducted to understand the behaviour of the subjects during the staircase force task, in which prolonged exposure to intraneural stimulation is required. For this reason, we were only interested in tracking the intensity of elicited sensations^20^ and in the relative rather than in the absolute adaptation times. The risk of overestimation of the decay time constants was minimized by asking the subject to keep the attention on the stimulation. We believe the consistency of the results indicates that the method was in any case robust. However, this is a potential limitation of the study, which we will address in future experiments.

Two factors leading to the differences between the two modulation strategies, which ultimately led to a better performance in functional tests, were the different sensitivities and adaptation times. The reasons for these differences have only been hypothesized in this work. Both experimental tests and modelling analyses will be performed in the future to understand the dynamics of adaptation in the two cases. Experimental tests will be broadened to guarantee a complete investigation of the phenomenon: adaptation occurring when stimulation trains of varying charge or frequency are delivered, and time of recovery of the sensations will be observed.

Finally, it would be of great interest to repeat this study with non-invasive sensory feedback approaches (e.g., TENS^42^).

## Materials and Methods

### Subject recruitment

No statistical methods were used to predetermine the sample size. Two left trans-radial amputees were involved in the clinical investigation. The first subject (Subject 1) was a 48-year-old female with a traumatic trans-radial amputation of the distal third of the left forearm (her dominant hand), which occurred 23 years before her enrolment in the trial. She was implanted in June 2016. The second subject (Subject 2) was a 53-year-old female trans-radial (proximal third of the forearm) amputee. The amputation occurred in December 2015, following a traumatic accident at work. In July 2017, Subject 2 received the same neural implants as Subject 1. Each subject was enrolled for a period of approximately 6 months, during which experimental sessions were randomized. The data reported in this manuscript were obtained over a period of several days with both amputees. Passive stimulation tasks were performed over the whole period of the clinical trial for both subjects, excluding the first week after surgery, in which the subjects rested. Subjects performed tests with intraneural stimulation twice a week (8 hours per specific experiment with periodic pauses (15 minutes) to avoid fatigue. The passive stimulation tasks sequences were randomized over time for both subjects. The functional tasks, a part for SFCT, were performed in two different sessions separated by a one-month interval (Figure S5) to evaluate the effect of training in prosthesis use over time.

Ethical approval was obtained by the Institutional Ethics Committees of Policlinic Agostino Gemelli at the Catholic University, Rome, Italy, where the surgery was performed. The protocol was also approved by the Italian Ministry of Health. Informed consent was signed. During the entire length of our study, all experiments were conducted in accordance with relevant guidelines and regulations. Informed consent for publication of identifying information/images was signed.

### Surgical procedures

The surgical approach to implant TIMEs is extensively reported elsewhere^7^. Briefly, during general anaesthesia, through a 15-cm-long skin incision on the left arm, the median and ulnar nerves were exposed to implant a proximal and a distal TIME in each nerve (scheme in Fig. 1). Each TIME had 14 active sites usable to deliver the electrical stimulation. Stimulation pulses were delivered via percutaneous wires. After 180 days, under an operating microscope, the four microelectrodes were removed, in accordance with the protocol and the obtained permissions.

### Sensation characterization

After the implantation, each channel of all the electrodes was connected to a stimulator used to drive the stimulation of TIME electrodes (Ripple LLC, USA). The stimulator delivered 2-sec trains of electrical current of variable amplitude, duration, and frequency. The sensation characterization (or mapping) procedure was performed (Figure S8), which allowed us to explore the subjects’ sensation related to the stimulation from different electrodes and active sites. This procedure was repeated for amplitude and frequency modulation, to find the difference in evoked sensations between the two strategies in terms of quality, location, extent, type and intensity. For this study, two active sites for each subject’s implanted nerve were chosen and explored using both modulation strategies. In the characterization procedure, the prosthesis was disconnected, and the subject was connected only to the neurostimulator using the transcutaneous cables of the implanted TIMEs. The stimulation was triggered by the experiment using a custom-made MATLAB program in an ‘open-loop’ (or passive) setup. The subject was blind to the stimulation configuration delivered.

Two-second trains of current pulses with variable amplitude or repetition frequency were delivered at least 5 times through every active site. Charge-balanced, biphasic, cathodic-first, rectangular stimulation pulses were applied versus a ground electrode integrated on the TIME. For the amplitude modulation, the pulse amplitude varied between 10 μA and 980 μA (steps of 10 μA), while the pulse width (from 10 μs to 120 μs) was fixed, as was the train frequency (50 Hz, as in^7^). For the frequency modulation, the pulse amplitude (from 10 μA to 980 μA, steps of 10 μA) was fixed, as was the pulse width (from 10 μs to 120 μs, with steps of 10 μs), at the values eliciting the subjects’ perceptual threshold (at 50 Hz). The pulse frequency was modulated by increasing from 1 to 1000 Hz in steps of 5 Hz. In the sensation characterization procedure, we increased the amplitude (for LAM) or frequency (for LFM), step by step, to give the subject the time to understand and to answer (i.e., in the case of threshold perception). The stimulation train lasted 2 sec with the same parameters, then it was increased by the minimum step, after a pause of 2 sec. The ramp continued until reaching the saturation value reported by the subject (as a level below the pain threshold).

Subjects were asked to report the location, extent, type and strength (on a scale between 0 and 10) of the generated percepts whenever they perceived them. They were also asked to describe the pleasantness and naturalness of the sensations on a scale between 0 and 5 at the perceptual threshold (not taking into account potential dependence on the intensity of the sensation). Using these data, it was possible to estimate the lower (thresholds) and upper (saturation) limits of the current amplitude able to induce sensations (defined, respectively, as the lowest stimulus pulse charge at which the subject reliably felt a sensation and the pulse charge at which the sensation became close to uncomfortable or painful, without reaching such a level, Figure S1 and S2)^43^.

Thus, a map of the sensations referred to the corresponding active sites was obtained and used for the calibration of the sensory feedback restoration system. The subjects could select a word to describe the evoked sensation, but they could also add a new sensation to the list in case of lack of a correct descriptor for the elicited sensation.

We tested the full range of parameters we had available in our hardware. However, the available range did not affect the characterization results. Indeed, in both the LFM and LAM, the saturation level (sub-pain intensity of the evoked sensations) was reached before or at 1000 μA and 1000 Hz (Figure S7).

### Sensitivity evaluation

The procedure used for determining the intensity levels was the same as the mapping procedure in which the subject had to report only changes in the sensation intensity (with a number between 1 and 10) instead of just the minimum and the maximum level. The modulation ramps were performed with LAM and LFM, randomly chosen, using the same stimulation ranges as in the sensation characterization paradigm. The subjects were blind to the experimental condition.

### Adaptation evaluation

To evaluate the time at which the evoked sensation ceased to be perceived by the subject, we designed the following protocol. A train of pulses of constant amplitude, duration and frequency was delivered. The subjects were asked to report the exact moments at which the sensation decreased and then disappeared. Three different amplitudes and frequencies were tested. According to the mapping procedure, we chose the frequencies and amplitudes that elicited perceptual threshold, a medium intensity and the maximum intensity of the sensation reported by the subjects. These values were in the order 10–50 Hz, 200–350 Hz, 700–800 Hz, 100–200 μA, 250–350 μA, 400–500 μA and for Subject 1; and 10–50 Hz, 150–250 Hz, 400–500 Hz, 200–300 μA, 350–450 μA, and 500–600 μA for Subject 2. The range was due to the different active sites that were used. The test was repeated 10 times for each of the four active sites in both conditions (i.e., the variation of the frequencies and the amplitudes).

The stimulation parameters to characterize adaptation were chosen on the basis of the subjects’ range of responses to stimulation itself. Indeed, since the performance of afferent nerve stimulation varies from subject to subject and from nerve interface to nerve interface, it is not possible to provide absolute knowledge about a specific range of stimulation parameters. Conversely, the minimum and maximum perceived sensations can be identified in every subject. This makes our results more general and applicable to different nerve stimulation techniques.

We asked the subjects to focus their attention on the sensory feedback intensity. They had to report to the experimenter as precisely as possible whether and when they perceived any change in the evoked sensation in terms of intensity (neglecting sensation type, location and quality). During the task, subject was alone with the experimenter and without sources of noise.

### Bidirectional prosthesis

Subjects were fitted with a bidirectional prosthesis, allowing control of hand opening and closing by processing surface electromyographic (sEMG) signals, and providing sensory feedback by means of electrical stimulation of the peripheral nerves. A robotic hand with pressure sensors integrated within each finger (IH2 Azzurra, Prensilia, Italy) was controlled using a custom, multithreaded C++ software program running on a Raspberry Pi 3 single-board computer (Raspberry Pi Foundation, UK). A recording and stimulating device (Neural Interface Processor, Ripple LLC, US) was also connected to the central single-board computer, acquiring sEMG data from four bipolar channels and providing stimulation outputs to the four neural electrodes. The instance of acquisition, recording and encoding lasted 100 ms.

Custom-moulded sockets were built with integrated screws to easily fix the robotic hand on the end. Holes were drilled in it to allow for the placement of sEMG electrodes on the stump.

### Prosthesis control

For prosthesis control, for both subjects, a 3-class (open, close, rest) KNN (k = 3) classifier was used^44^. Four bipolar channels of sEMG were acquired from forearm residual muscles, where palpation was used to place the electrodes in the optimal positions. The sEMG data were acquired with a sampling frequency of 1 kHz and filtered using a passband 4^th^-order Butterworth IIR filter between 15 and 375 Hz, as well as a notch to remove 50 Hz power hum and the harmonics at 100 Hz and 150 Hz. The waveform length, computed over a window of 100 ms for each channel, controlled the hand actuation speed (proportional control). The classifier ran every 100 ms.

### Prosthesis sensory feedback and modulation strategies

Two force sensors embedded in the little and index fingers of the prosthesis were used as control inputs for the intraneural stimulation of two active sites in the sensory innervation territories of median and ulnar nerves. In the amplitude modulation condition, the amplitude of biphasic, symmetric, cathodic-first and square pulses was modulated by the following linear relationship:

$$\begin{array}{llll}c & = & 0, & {\rm{when}}\,s < {s}_{0};\\ c & = & ({c}_{max}-{c}_{min})\ast (s-{s}_{0})/({s}_{75}-{s}_{0})+{c}_{min}, & {\rm{when}}\,{s}_{0}\le s\le {s}_{75};\\ c & = & {c}_{max}, & {\rm{when}}\,s > {s}_{75};\end{array}$$

where:

*c* is the amplitude of stimulation current,

*s* is the sensor readout,

*s*_0_ and *s*_75_ represent the minimum and maximum range of the sensor readout, respectively, which characterize the first contact of the robotic hand with an object and a value tuned to exploit the full range of sensations for all objects. *c*_*min*_ and *c*_*max*_ are the stimulation current amplitudes that elicited, respectively, the minimum and the maximum (i.e., below pain threshold) touch sensations, as reported by the subject according to the last sensation characterization procedure. The frequency of the stimulation was 50 Hz^7^. For the frequency modulation, the amplitude was fixed at the value that elicited the minimum touch sensation and the frequency was proportional to the sensor readout following the same rule as amplitude modulation. The overall sensory-motor control scheme and the different encoding strategies are provided in Fig. 1. The used ranges of charges and frequencies are in Figure S6.

### Functional Tasks

Five sensorimotor tasks were performed by the subjects: *Object Location Task* (OLT^7^), *Shape Recognition Task* (SRT^7^), *Compliance Recognition Task* (CRT^7^), *Force Control Task* (FCT^7^), *Staircase Force Control Task* (SFCT^7^). In all tasks, the subjects were acoustically shielded and blindfolded. They did not receive any systematic or prolonged training.

In this task (as presented in^20^), subjects were asked to grasp an object with the robotic hand and to recognize three positions in which a plastic cylinder was placed (ulnar, median zone or both). An experimenter positioned the plastic cylinder on the palm of the robotic hand. In the case of the median and ulnar zone condition, the cylinder was in contact with all the digits when the robotic hand was closed, while in the cases of ulnar or median zone conditions, the object was in contact with the last two digits or the first three, respectively. In the SRT, they had to distinguish three object shapes (ball, cylinder and trapeze) and in the CRT, they had to distinguish the stiffness of the object (foam cylinder, *soft*, and plastic cylinder, *hard*) relying only on the sensory feedback (amplitude or frequency).

In the FCT (Fig. 3A), as in^7^, the subjects were asked to apply 3 levels of self-selected grasping force (low, medium and high) on a dynamometer and to hold them for approximately 2 s. The participants were instructed to rely on the sensory feedback information to reproduce the three different levels of force. The patients performed a short familiarization session (approx. 5 minutes), during which they could squeeze the dynamometer with the bidirectional prosthesis exploring the sensory feedback. The velocity of the hand movement execution was randomly modified (three velocities) without informing the participants, in order to prevent that they could rely on learned closing times to execute the task. In each of these functional tasks, 90 trials were performed by each subject in each feedback condition. The FCT was executed with the prosthesis unmounted. The prosthesis and the dynamometer were constrained by two presses at a constant position, to guarantee the repeatability of the grasps.

In the SFCT, the setup was identical to the FCT. The participants needed to gradually increase the applied force, reproducing sequentially three different levels (low, medium and high) and then to gradually return to the baseline (high, medium and low). During the rising and descending phases (considered a force stair), they were instructed to maintain the indicated force level until feeling confident with the exerted level of force. In this task, 50 trials were performed for each subject in each feedback condition.

### Statistics and data analysis

All data were analysed using MATLAB (R2016a, The MathWorks, Natick, US). All statistics were performed using the available built-in functions. A one-sample Kolmogorov-Smirnov test was used to determine if the datasets associated with the various experiments were normally distributed. Since data were not fitted by a normal distribution, we used non-parametric alternatives (Kruskal-Wallis instead of ANOVA) and reported the average and standard deviation. All reported *p*-values resulting from the Kruskal-Wallis test measure the significance of the chi-square statistic. When appropriate, multi-group correction was applied using the Tukey-Kramer (*multcompare()*, MATLAB) test. Fisher’s exact test (*p*) was used to compare the performance using different encodings in the functional tasks.

For the analysis of the force levels, for each trial, the duration was normalized, and an average force value was computed over a fixed interval (60% to 90% of trial completion). To compute the performance score (given as a percentage of correct trials), we first obtained the average force value for each force level using the method outlined above. Then, we assigned each repetition to the nearest force level. Finally, we computed the performance score as the percentage of repetitions correctly assigned to the right force level. The number of repetitions for each experiment is reported in the corresponding figure captions.

## Electronic supplementary material

Supplementary information

## Data Availability

The datasets generated during and/or analysed during the current study are available from the corresponding author on reasonable request.
