# Supplementary material for: Comparison of linear frequency and amplitude modulation for intraneural sensory feedback in bidirectional hand prostheses
Source: Sci Rep. 2018 Nov 12;8:16666. doi: 10.1038/s41598-018-34910-w (PMC6232130; doi:10.1038/s41598-018-34910-w)
Supplement: Supplementary file 1 — Supplementary information [file 41598_2018_34910_MOESM1_ESM.docx]

**Comparison of linear frequency and amplitude modulation for intraneural sensory feedback in bidirectional hand prostheses**

G. Valle^1,2,+^, F. M. Petrini^1,5,+^, I. Strauss^1,2,+^, F. Iberite^2^, E. D’Anna^1^, G. Granata^3^, M. Controzzi^2^, C. Cipriani^2^, T. Stieglitz^4^, P. M. Rossini^3^, A. Mazzoni^2&^, S. Raspopovic^1,5&^, S. Micera^1,2,*^

^1^Bertarelli Foundation Chair in Translational Neuroengineering, Centre for Neuroprosthetics and Institute of Bioengineering, School of Engineering, École Polytechnique Fédérale de Lausanne (EPFL), Lausanne, Switzerland.

^2^Center for Neuroscience, Neurotechnology, and Bioelectronic Medicine and BioRobotics Institute, Scuola Superiore Sant'Anna, Pisa, Italy.

^3^Institute of Neurology, Catholic University of The Sacred Heart, Policlinic A. Gemelli Foundation, Roma, Italy.

^4^Laboratory for Biomedical Microtechnology, Department of Microsystems Engineering–IMTEK, Bernstein Center, BrainLinks-BrainTools Cluster of Excellence, University of Freiburg, Freiburg D-79110, Germany.

^5^Department of Health Sciences and Technology, Institute for Robotics and Intelligent Systems, ETH Zürich, 8092 Zürich, Switzerland

^+^Equally Junior Contributors

^&^Equally Senior Contributors

^*^Corresponding Author: Silvestro Micera ([silvestro.micera@epfl.ch](mailto:silvestro.micera@epfl.ch)[santannapisa.it])

**SUPPLEMENTARY MATERIALS**


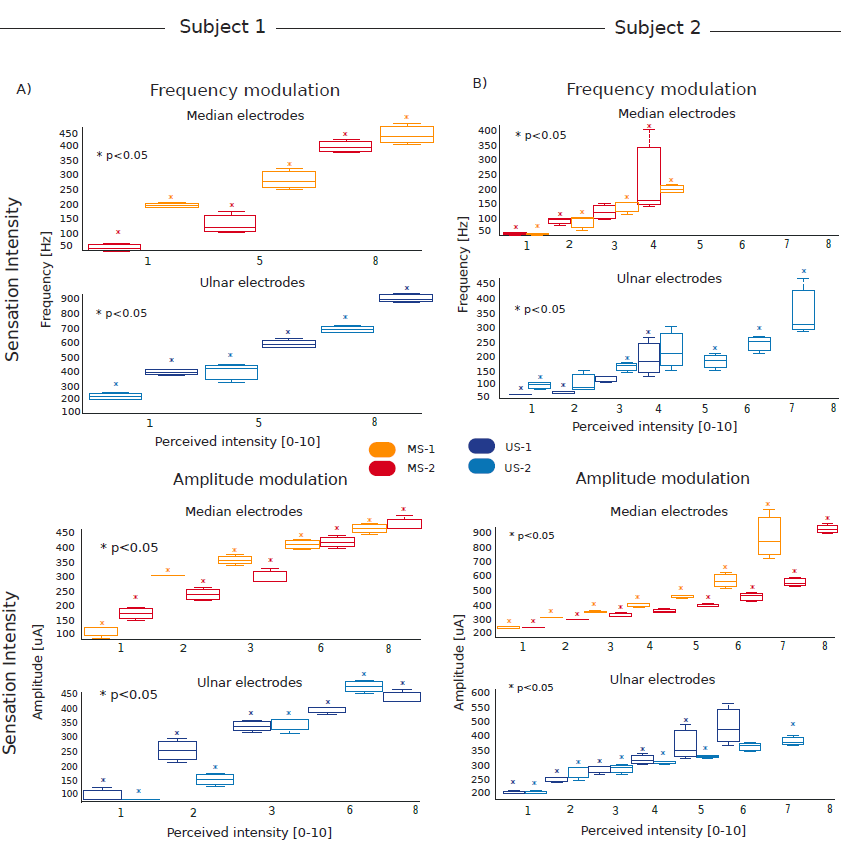


**Figure S1. Statistics on the pressure levels perceived.** The box plots show the range of all amplitudes and frequencies for each pressure level perceived by Subject 1 (B) and Subject 2 (C) related to each AS used. Data in the figure is represented as median and 25^th^-75^th^ percentiles. * means p < 0.05. Two-tailed Kruskal-Wallis test with Tukey-Kramer correction for multiple groups of data was performed. N= 5 repetitions x 4 AS=20 per each stimulation condition (LAM and LFM).


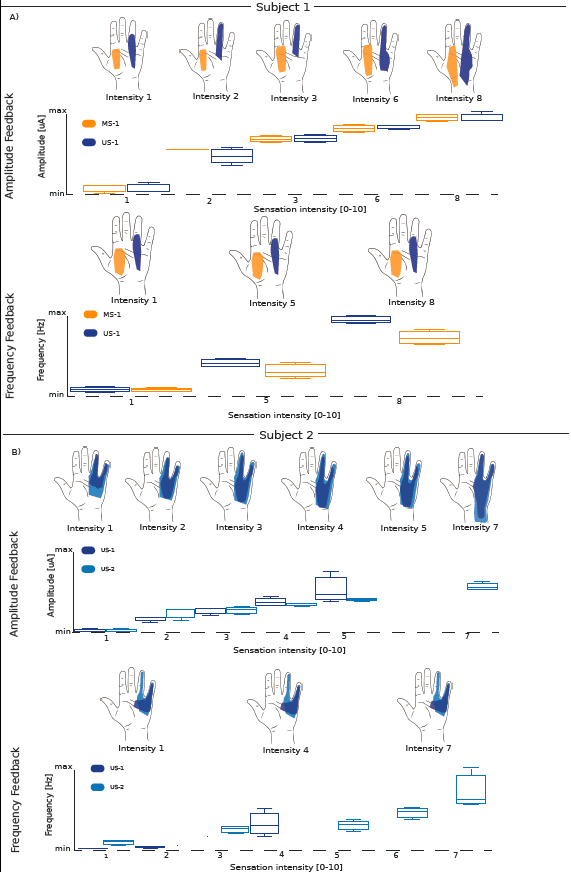


**Figure S2. Perceived sensation intensities.** Sensation locations and intensities reported by Subject 1 (A) and Subject 2 (B) during the modulation of amplitude and frequency of the stimulation train are reported. In the maps of hand sensations, each colored area indicates the 75^th^ percentile of the occurrences of all the phantom sensation locations evoked for two active sites (one on median and one on the ulnar nerve) across all trails during the Sensitivity evaluation protocol (Methods) f. The box plots identify the range of amplitudes and frequencies; the central mark indicates the median, and the bottom and the top edges the 25^th^ and 75^th^ percentiles, respectively. Only the statistically different levels are shown (with p<0.05, Kruskal-Wallis test with Tukey-Kramer post-hoc test). N= 5 repetitions x 4 AS=20 for each stimulation condition (LAM and LFM). Dashed x-axis indicates a non-linear x scale.


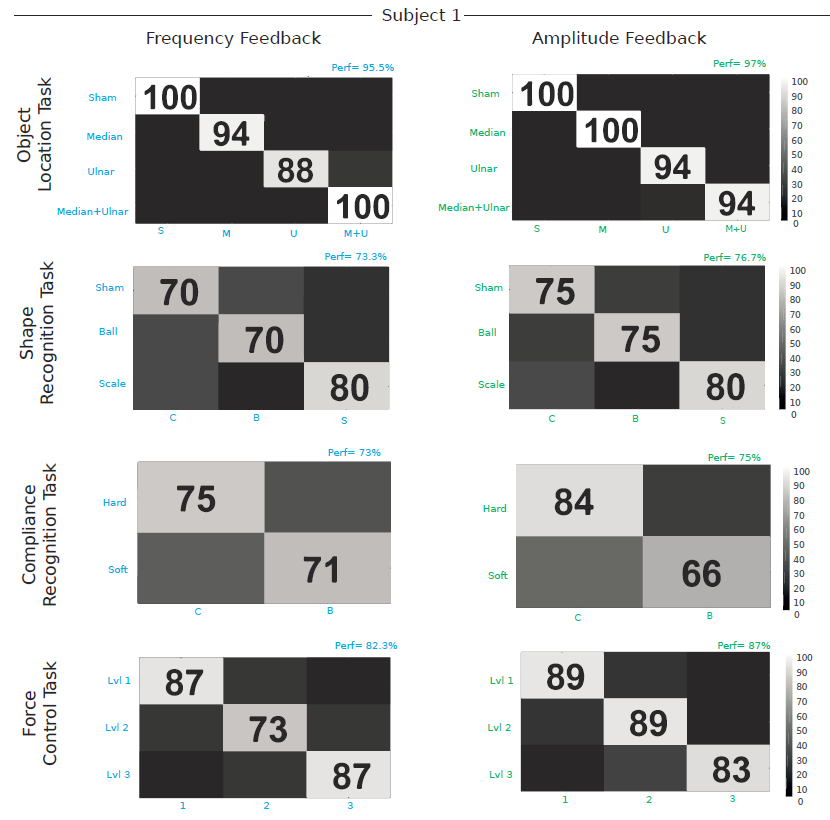


**Figure S3. Extended performance in the functional tasks for Subject 1.** Confusion matrices reporting the performance for each task for Subject 1 are shown. For each matrix, the mean performance is reported. N=90 repetitions per each stimulation condition (LAM and LFM).


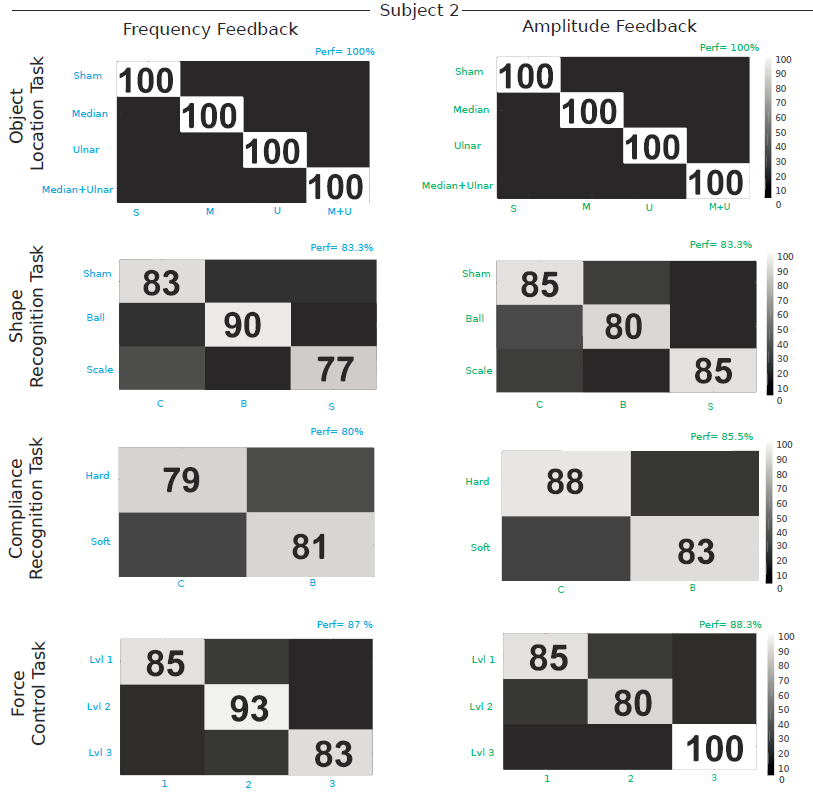


**Figure S4. Extended performance in the functional tasks for Subject 2.** The confusion matrices reporting the performance for each task for Subject 2 are shown. For each matrix, the mean performance is reported. N=90 repetitions per each stimulation condition (LAM and LFM).


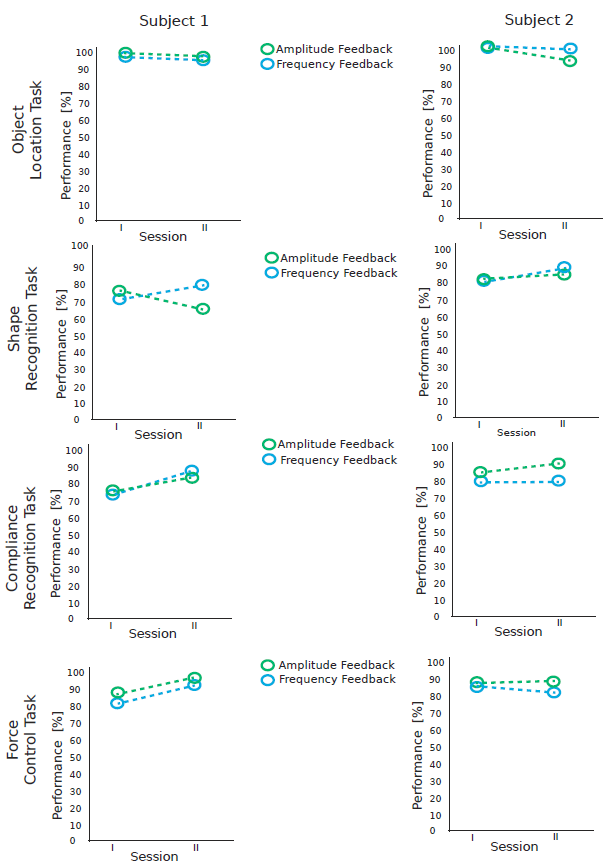


**Figure S5. Performance over time in the functional tasks for Subject 1 and 2.** Session I and II are executed at one month of distance (at months 2 and 3 after implantation for subject 1, and months 3 and 4 for subject 2). In between this period, both subjects used the bidirectional prosthesis twice a week. The average performance is shown using both encoding strategies (color coded) in every functional task. In each session, N=90 repetitions for every task and subject are performed. Fisher test was used to test the difference between Session I and II. No statistical difference in the performance over time was found (p>0.05).


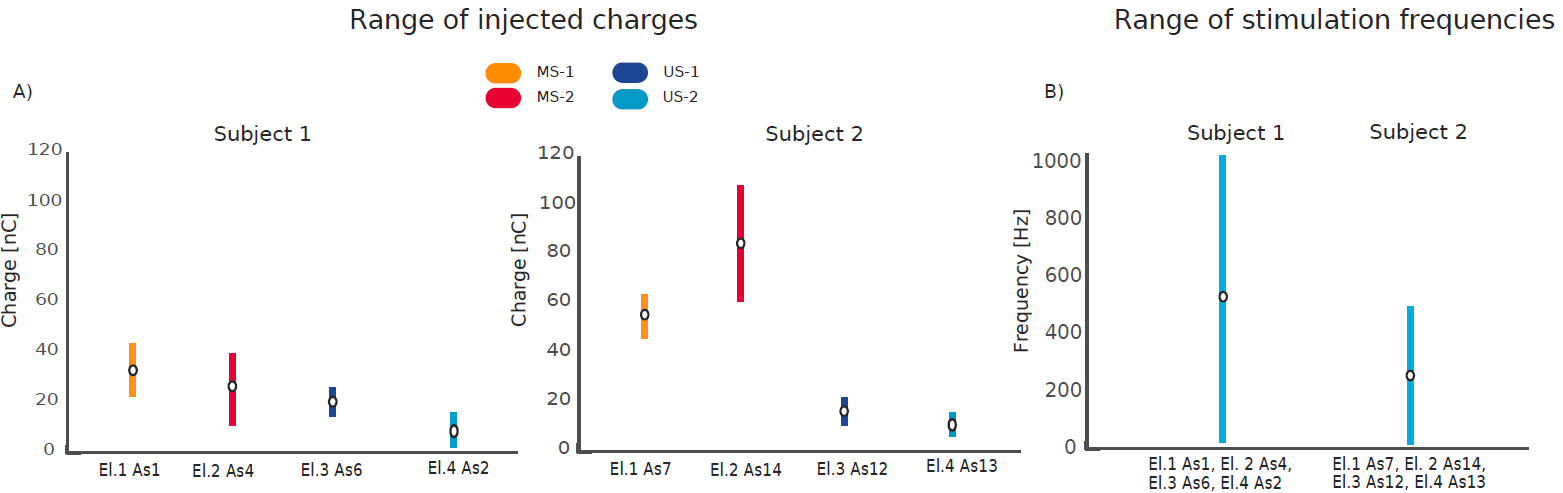


**Figure S6. Stimulation charge and frequency ranges.** The range (min-max) of charge, which has been injected for each AS used during the functional tasks, is reported for both subjects when they used LAM (A) or LFM (B). (B) Each AS has a range of 1Hz-1kHz and 1Hz-500Hz for Subject 1 and Subject 2 respectively.


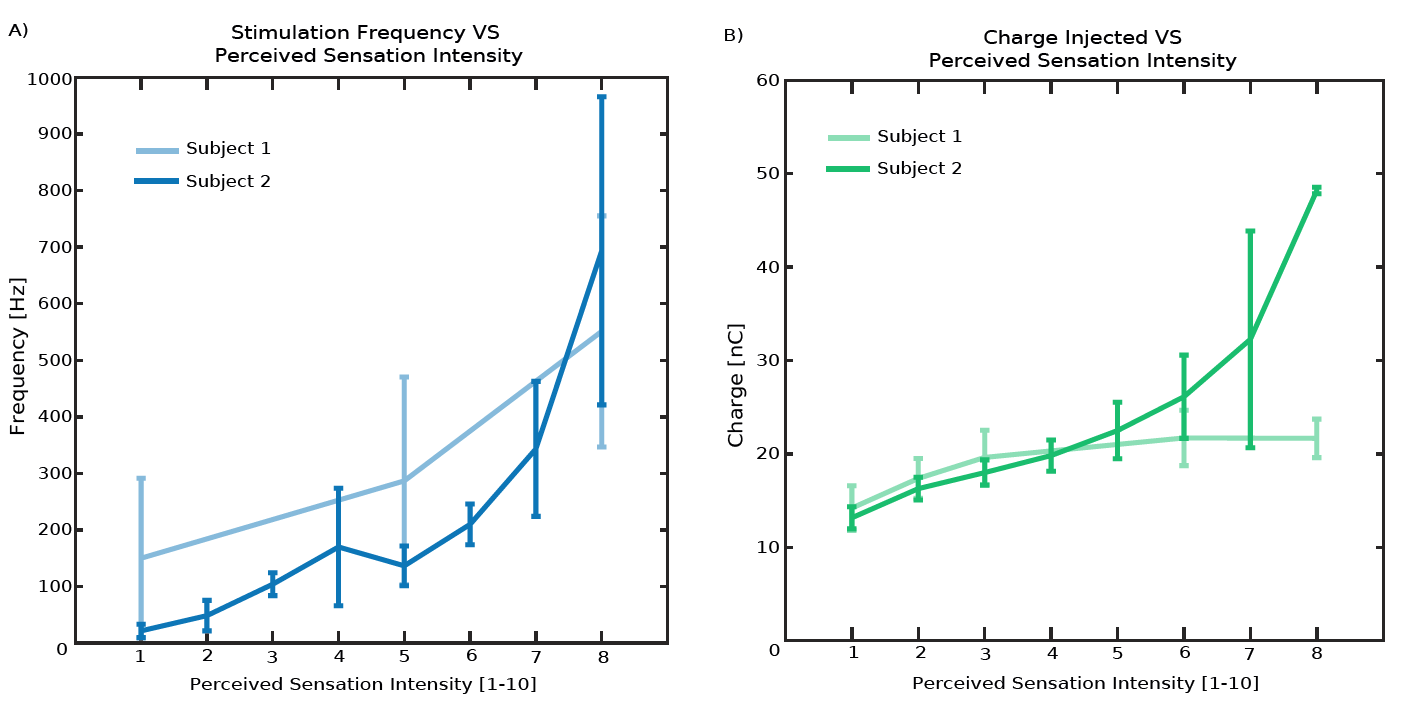


**Figure S7. Stimulation parameters and sensation intensity.** Stimulation frequency (**A**) and charge (**B**) as a function of perceived sensation intensity (all other stimulus parameters held constant). The data are related to the tested AS (Figure 2) and both subjects. Error bars denote the standard deviation. N=5 repetitions x 4 AS=20 per each stimulation condition (LAM and LFM).


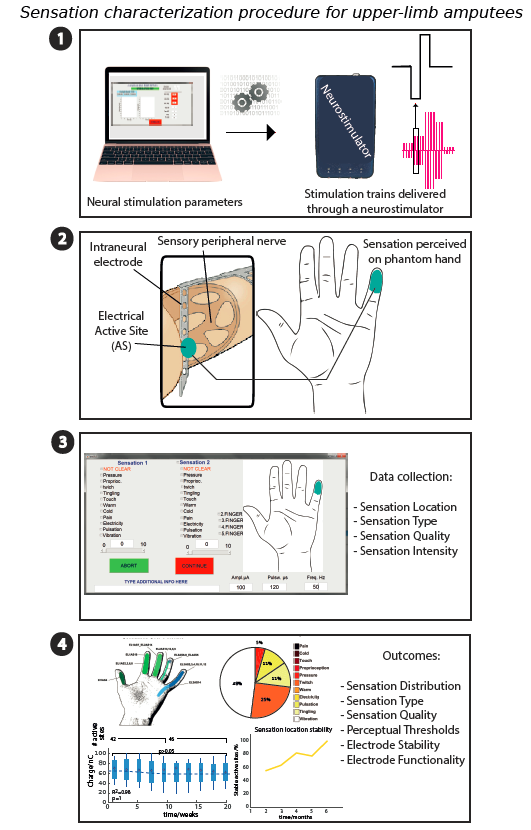


**Figure S8. Sensation characterization procedure.** (1) Stimulation parameters are selected. The stimulation trains are delivered using the neurostimulator. (2) Patient perceives stimulation-evoked sensation on the phantom hand thanks to the neural implant (3) A custom GUI allows patients to report any sensation properties. (4) Experimenters collect all sensation characterization outcomes.


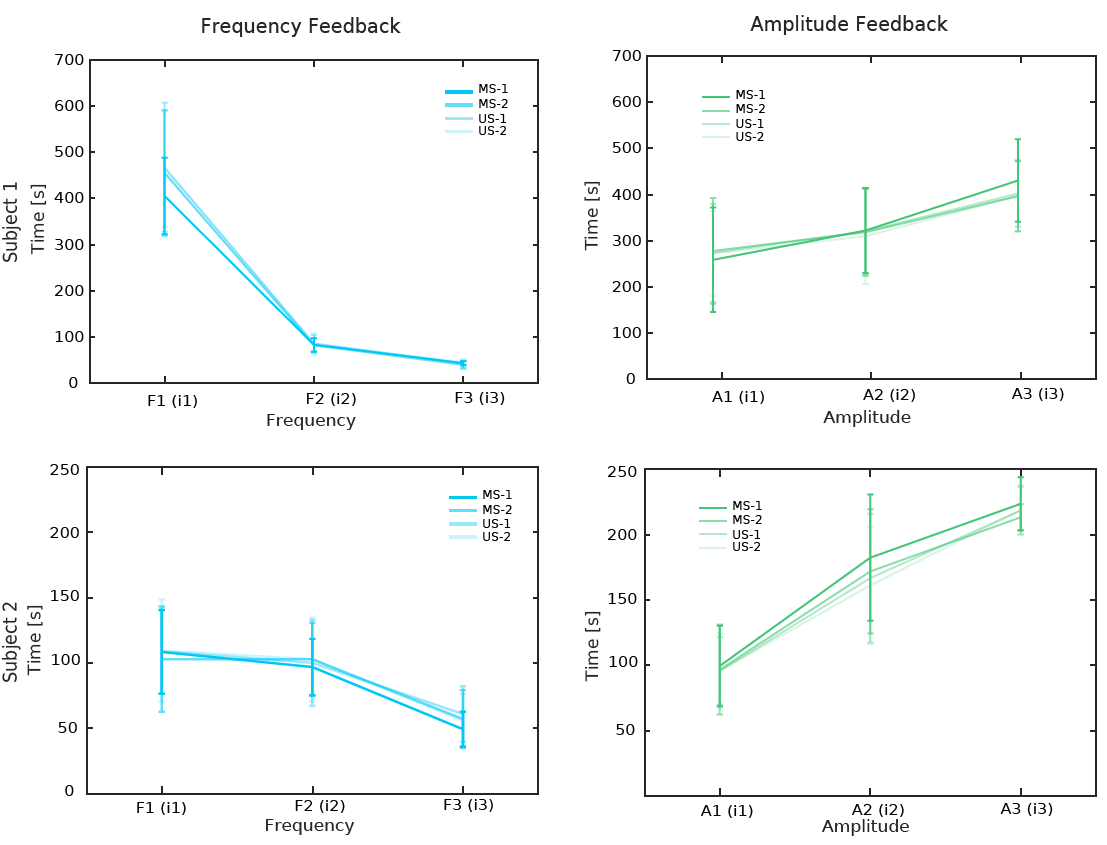


**Figure S9. Adaptation time broken down by active sites.** The adaptation time is shown at different frequencies and amplitudes of the stimulation trains and showed for each AS. A1, A2, A3 or F1, F2, F3 are the amplitudes or frequencies at which the subjects reported respectively perceptual threshold (i1), medium (i2) and high (i3) intensity of the sensation perceived during the mapping procedure. The data in the figure is represented as mean ± std. N=5 repetitions x 4 AS for each stimulation condition (LAM and LFM).


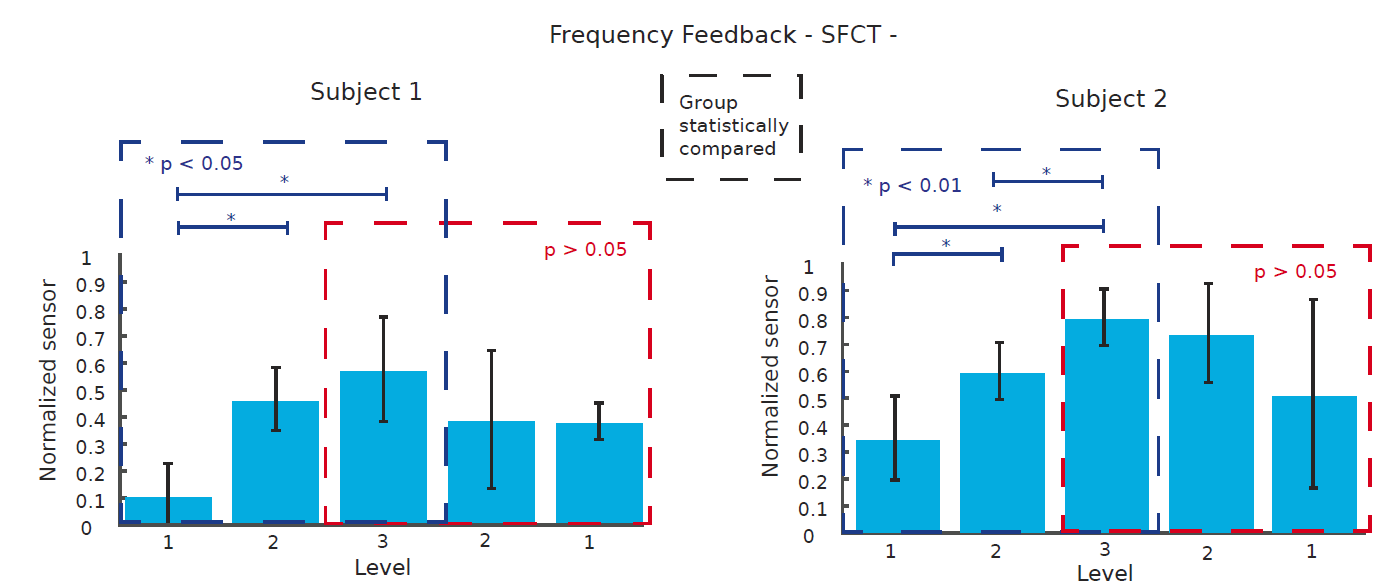


**Figure S10. SFCT broken down by force phase during LFM.** Statistical analysis considering separately rising (blue) and descending (red) phases is shown. The force output is presented for the robotic hand finger during SFCT with LFM. A Kruskal-Wallis test with a Tukey-Kramer post-hoc test for multi group comparison (grouped by force phase, dashed line) was performed over all the single step force trials (staircase up-staircase down). N=5 levels x 10 repetitions x 2 conditions (LAM and LFM) for Subject 1 and Subject 2. The test shows significantly different levels, achieved in terms of the maximum force-per-level reached, in the rising phase only. The data in the figure are represented as means ± std. * indicate p-value.


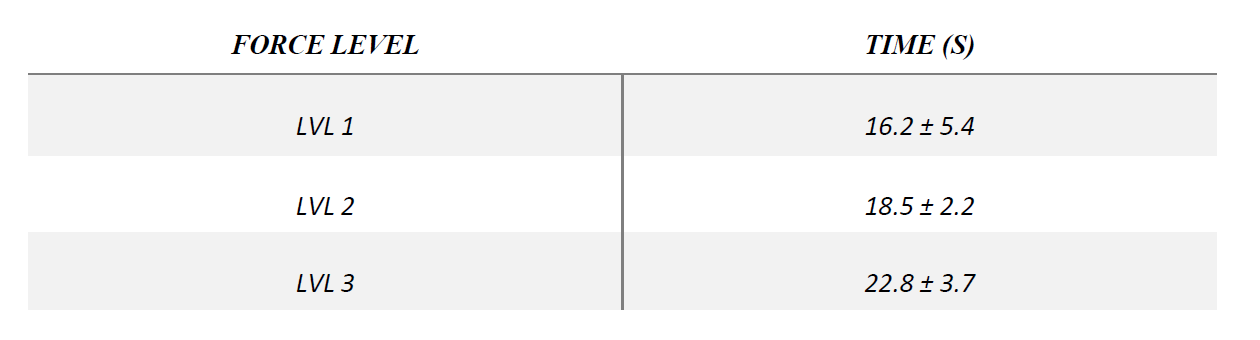


**Supplementary Table 1. Duration of force levels.** Time (seconds) needed by the subjects to reproduce a specific level of force. Data are related to FCT (N=6 repetitions). Mean ± standard deviation is reported for each level.
